# Combined Radial and Ulnar Epiphyseal Injury at the Wrist: A Rare Case

**DOI:** 10.7759/cureus.11843

**Published:** 2020-12-02

**Authors:** Thirumalesh Krishna Reddy, Raghavendra Kosagi Sharaph

**Affiliations:** 1 Orthopedic Surgery, Aster CMI Hospital, Bangalore, IND; 2 Orthopedics, Aster CMI Hospital, Bangalore, IND

**Keywords:** wrist epiphyseal injury, salter harris type 2, radius ulna combined epiphyeal injury, epiphyseal injury, manipulation under anesthesia, k-wire

## Abstract

Epiphyseal injury involving both the radial and ulnar epiphysis at the wrist is rare and presents a diagnostic and therapeutic challenge. We report a case of a young adolescent female who presented with such an injury after a fall. She had a Salter-Harris Type 2 epiphyseal injury involving the distal end of both the radius and ulna. She was treated with gentle manipulation under anesthesia and stabilization with K-wires. We recommend early reduction with gentle manipulation for these injuries. The case is presented for its rarity and we have described our preferred treatment. Complications of these injuries involve growth disruption with possible length discrepancy between the radius and ulna, deformities, injury to the triangular fibro-cartilagenous complex and rarely median neuropathy.

## Introduction

Injuries to the wrist joint are common in children. The commonest epiphyseal injury around the wrist is to the distal radial epiphysis. The injuries to the growth plate were originally classified by Salter and Harris in 1963 [1]. 73% of all epiphyseal injuries are type 2 injuries [2]. We present a case of injury in a 12-year-old girl, with a type 2 epiphyseal injury to both the distal radius and distal ulna, diagnosed on standard wrist radiographs. We did a thorough search in PUBMED, Google Scholar and Google search engine for similar reports. We found many articles describing injuries to the distal radial epiphysis in isolation and some with associated ulnar physeal injuries. There was only one case report of combined injury with a type 2 injury to the radius epiphysis and a type 4 injury to the ulnar epiphysis [3].

## Case presentation

A 12-year-old right-hand dominant girl, presented to the emergency with a history of fall on outstretched hand. She complained of pain, swelling and a deformity of the right wrist. Active movements of the wrist were painful.

**Figure 1:**
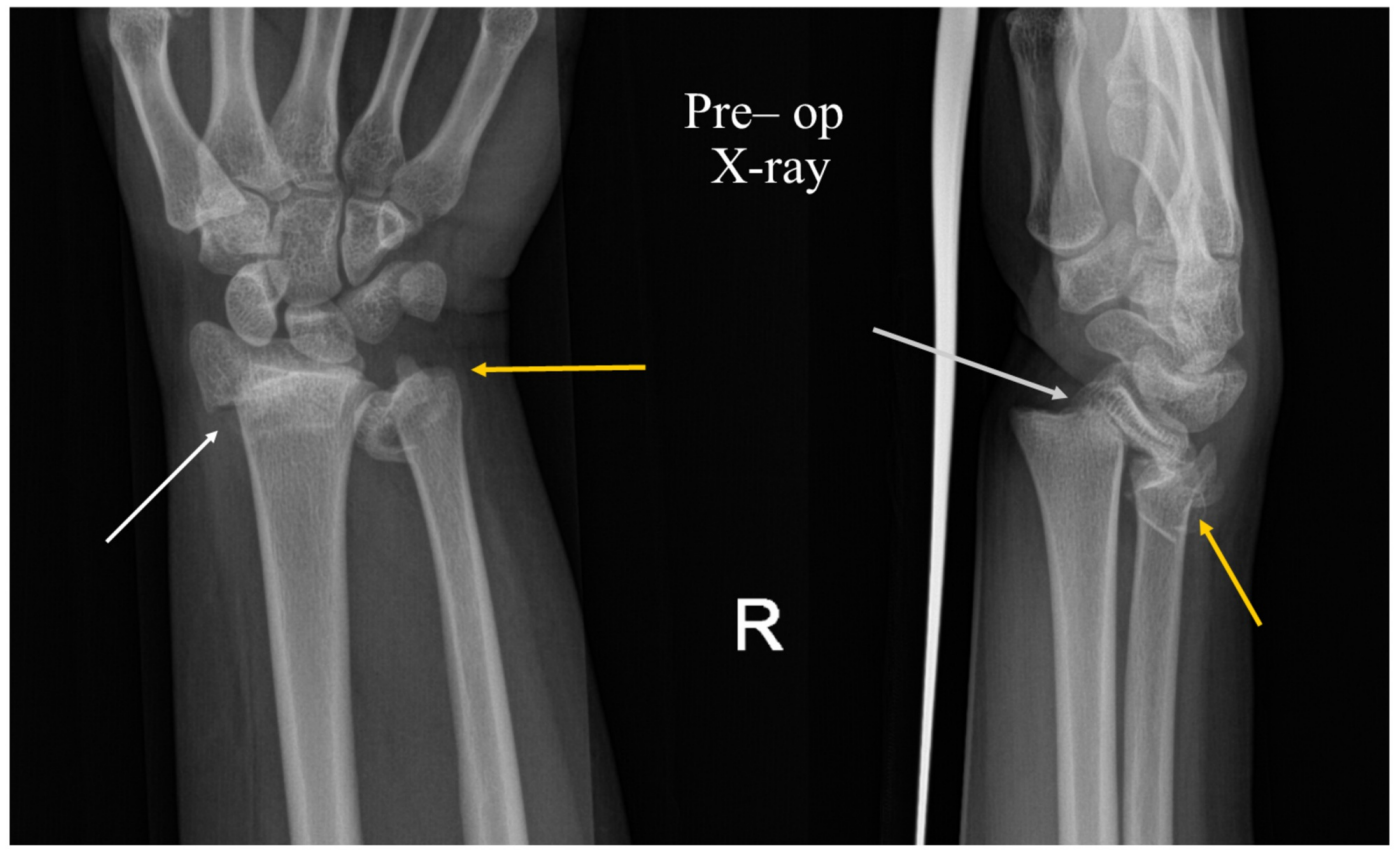
Pre-operative radiographs of the right wrist (antero-posterior and lateral views) White arrows: radial physeal injury; yellow arrows: ulnar physeal injury.

**Figure 2:**
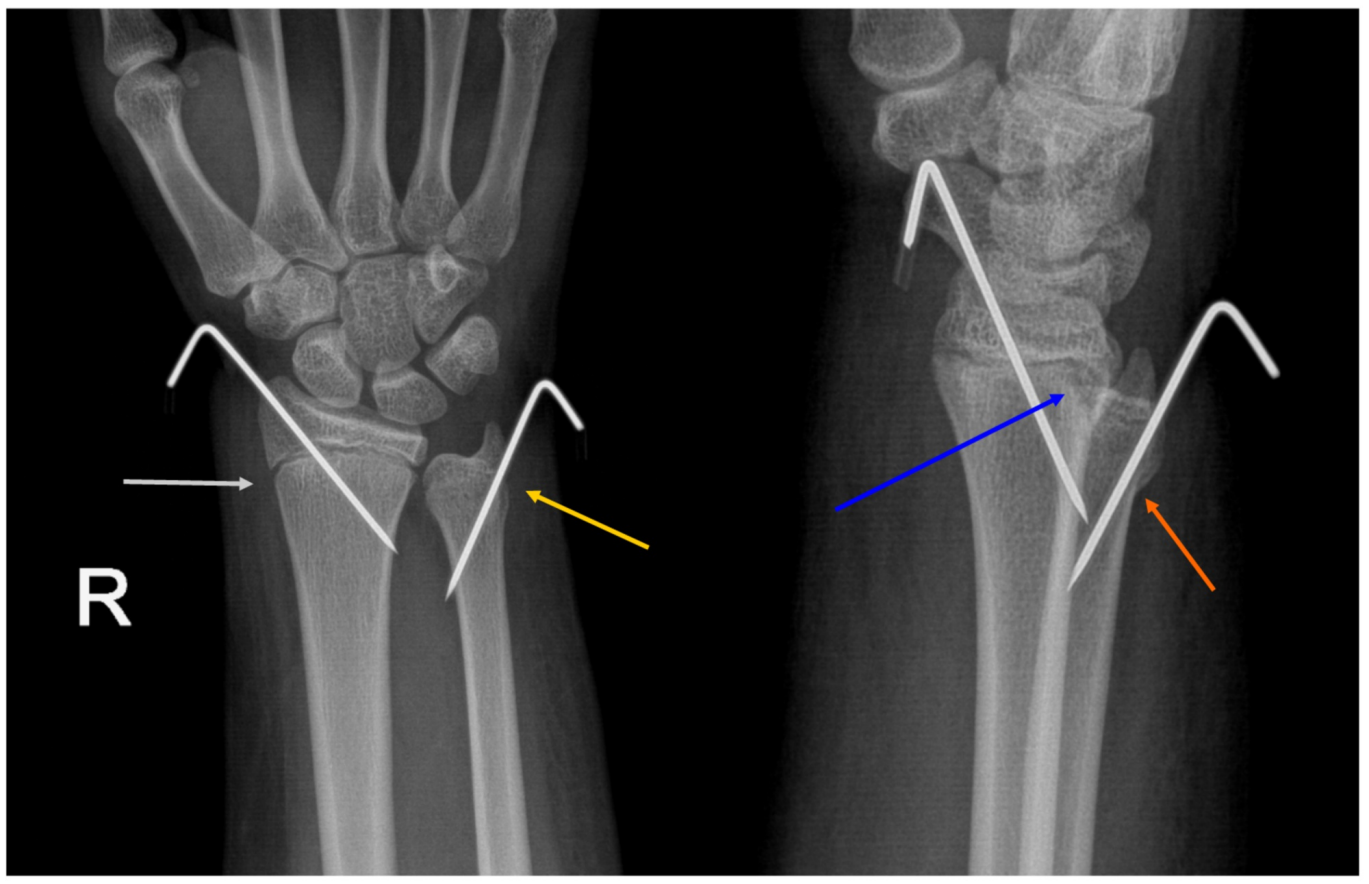
Post-operative radiographs of the right wrist showing K-wires in situ White arrow: radial physis, post reduction; yellow arrow: ulnar physis, post reduction; blue arrow: fleck sign in the radius; orange arrow: fleck sign in the ulna.

Clinical examination revealed a swelling and deformity of her right wrist. Wrist movements were limited by pain. Clinically, there were no injuries to the neurological or vascular structures. There were no external injuries. Standard antero-posterior and lateral radiographs of the wrist were performed. The X-rays showed physeal injuries to both the distal radius as well as the ulna (Figure 1). Near-total displacement in the dorsal direction was noted. It was initially graded as Type 1 Salter-Harris injury, but post reduction X-rays showed a clear metaphyseal involvement similar to a type 2 injury (Figure 2).

The patient was taken up for closed reduction on the same day. Under anesthesia, the epiphyseal fracture was reduced with longitudinal traction. Minimal and gentle manipulation was done to reduce both the radial and ulnar epiphysis. The ulnar epiphysis needed gentle manipulation. The ulnar epiphysis was reduced with gentle extension and traction, followed by palmar pressure. C-arm images were acceptable. The fluoroscopic images showed instability in the ulnar fracture. Both the fractures were stabilized with smooth 1.2 mm K-wires. The arm was protected in an above elbow plaster slab. At the time of submitting this article, the patient was two weeks post-operation. The post-operative period has been uneventful.

## Discussion

Injuries around the wrist are common among children. The injuries to the radial epiphysis are well documented. There are very few documented reports of isolated ulnar epiphyseal injuries as they are very rare [4,5]. Most epiphyseal injuries around the wrist are Salter-Harris Type-2 injuries. A review of the literature shows that closed reduction of these injuries with or without K-wire fixation is adequate in most cases. Studies have shown that one should avoid repeated closed manipulations or manipulation after seven days of the initial injury [6]. The reduction of the ulnar physeal injury may sometimes be hindered by the interposition of the extensor carpi ulnaris tendon [7]. In such cases, open reduction is recommended. In unstable injuries, internal fixation with smooth pins are recommended. The implant and the plaster are retained for at least four weeks after which they are removed and the limb mobilized.

Most studies report good post-operative outcomes. However, studies show that complications are to be expected and the parents to be counseled adequately regarding the same. Complications of radial physeal injury include malunion, length discrepancy due to partial or complete physeal arrest and rarely acute median neuropathy and compartment syndrome. Symptomatic malunions may be managed with corrective osteotomies. Radial and ulnar length discrepancy may be addressed with lengthening or shortening procedures at a later stage.

Complications of ulnar physeal injuries include premature ulnar physeal closure and ulnar shortening seen in 55% of patients [4]. Other complications are the angular deformities of the radius and ulna and length discrepancy of the two forearm bones. However, most patients were asymptomatic and only a few patients need surgery. The patient should also be assessed for injuries to the triangular fibro-cartilagenous complex. Walsh et al. reported that 4% of all wrist injuries presented with Galeazzi injuries [8]. Some authors have recommended routine CT and MRI scans for these injuries with high index of suspicion [3]. The anatomic reduction of the ulnar injury is paramount for the integrity of the distal radioulnar joint. Most patients with physeal injuries around the wrist have some degree of growth disruption with consequent deformity, but remain clinically asymptomatic.

## Conclusions

Combined injury of the distal radius and ulna is a rare clinical entity. Injuries of the ulnar epiphysis may be missed and need a high index of suspicion. We recommend early treatment with gentle manipulation and stabilization with K-wires when indicated. Patients and the parents must be adequately counseled about the various complications including growth disruption and resultant deformities, though most will remain asymptomatic.
